# Intrapersonal Variation in Goal Setting and Achievement in Health Coaching: Cross-Sectional Retrospective Analysis

**DOI:** 10.2196/jmir.8892

**Published:** 2018-01-26

**Authors:** Anne M Wallace, Matthew T Bogard, Susan M Zbikowski

**Affiliations:** ^1^ Wellness Science and Analytics Humana, Inc Louisville, KY United States

**Keywords:** health coaching, health risks, chronic conditions, behavior change

## Abstract

**Background:**

Chronic conditions in the United States are among the most costly and preventable of all health problems. Research suggests health coaching is an effective strategy for reducing health risks including decreases in weight, blood pressure, lipids, and blood glucose. Much less is known about how and when coaching works.

**Objective:**

The aim of this study was to conduct an analysis of intrapersonal variations in participants’ progression in health coaching, examining gender and age-related differences.

**Methods:**

This was a cross-sectional, retrospective analysis of 35,333 health coaching participants between 2012 and 2016. Differences in number of goals and activities set and completed, and number of interactions were assessed using negative binomial models. Differences in goal type were assessed using logistic regression for gender and using the Welch test for age to account for unequal variances.

**Results:**

Participants choosing online coaching were more likely to be younger and female (*P*<.001). Gender and age differences were found for the types of goals set by participants. Regarding program activity, women set and completed 12% more action steps than men (*P*<.001), averaging 21% more interactions than men (*P*<.001); no gender differences were found in number of goals completed (*P*=.12), although the percentage of males and females completing goals was significantly different at 60 and 120 days postenrollment (*P*<.001). Results indicated significant age-related differences in all aspects of program activity: number of interactions, goals set and completed, action steps set and completed (all *P* values <.01), as well as significant differences in percentage of individuals completing initial goals within 30 days, with older individuals completing more than younger individuals did (all *P* values <.001).

**Conclusions:**

This study found significant intrapersonal variation in how people participate in and progress through a coaching program. Age-related variations were found in all aspects of coaching activity, from modality preference and initial choice of goal type (eg, weight management, tobacco cessation) to goal completion, whereas gender-related differences were demonstrated for all program activities except number of goals set and completed. These findings indicate that to maximize behavior change, coaches need to personalize the coaching experience to the individual.

## Introduction

Chronic conditions in the United States currently are among the most common, costly, and preventable of all health problems. As of 2012, approximately half of all adults—117 million people—had one or more chronic health conditions [1]. By 2021, according to the Centers for Medicare and Medicaid Services, health care spending will account for almost one-fifth of the gross domestic product [2]. Lifestyle behaviors such as an unhealthy diet, physical inactivity, and tobacco use are among the primary risk factors for disease onset.

Current literature suggests health coaching is an effective strategy for promoting health behavior change [3,4], including improving nutrition, increasing physical activity, and improving adherence to medications [5-7]. Health coaching’s effectiveness also has been demonstrated to reduce health risks, including decreases in body mass and weight loss [8-11], positive changes in blood pressure and lipid levels [5,12], and decreases in blood glucose and glycated hemoglobin A_1c_ [13-15]. Moreover, research demonstrates the effectiveness of health and wellness coaching in improving the health status of individuals with chronic conditions, most notably improved self-care regimen compliance [16].

Although the body of literature demonstrating the effectiveness of health coaching is growing, much less is known about how and when it works. Some research has found intrapersonal variation in coaching engagement and retention, including gender and age-related differences [17-19]; however, research has not yet addressed intrapersonal differences in how people set and make progress with goals as part of a coaching program.

Goal setting and achievement are foundational to the coaching process and to health behavior change more generally [20-23]. In particular, behavior change is enhanced via setting of goals that are SMART (specific, measurable, action oriented, realistic, and time bound), accompanied by and supporting short-term goals or action steps [24-29], Moreover, research highlights the importance of obtaining goal commitment as part of the goal-setting process in addition to ongoing monitoring of and review of goals in behavior change interventions [29-31]. For this reason, closer examination of intrapersonal variation in the process of goal activity within the context of health coaching can shed valuable light on how to support behavior change in a variety of different types of people.

In this study, we conduct a detailed analysis of intrapersonal variations in how participants engage in coaching and in their goal-related activities as they progress through a health coaching program, examining gender and age-related differences in the choice of coaching modality, the types of goals set by individuals, and the rate at which goals and supporting action steps are set and completed as participants progress through the program.

## Methods

This was a cross-sectional retrospective analysis of individuals enrolled in health coaching as part of an employer-sponsored wellness benefit or as part of wellness programming bundled into an individually purchased health insurance plan. All personally identifiable data were gathered and prepared for analysis following organizational, regulatory, and Institutional Review Board (IRB) policies and practices. The study received IRB approval from Schulman IRB, Cincinnati, OH, on December 12, 2016.

### Sample

The sample was comprised of 35,333 individuals aged 18 years or older enrolled in the coaching program between January 2012 and August 2016, and who set one or more goals with their coach. Females comprised the majority of participants, making up 26,778 (75.79%) of the sample. Males comprised 8493 (24.04%) of the sample; the gender of 62 (0.18%) participants was unknown. The age breakdown was as follows: 4653 (13.17%) of participants were younger than 30 years, 18,106 (51.24%) were between 30 and 50 years, 8663 (24.52%) were between 51 and 64 years, and 3911 (11.07%) were 65 years and older.

### Intervention

The objective of the coaching program was to reduce health-related risks. Participants could choose to work on one or more health-related areas including weight management, tobacco cessation, healthy eating, fitness, stress management, cholesterol management, diabetes management, blood pressure management, or back care. Participants enrolled in coaching could remain active in the program as long as they were eligible through an employer-sponsored or individual benefit.

Health coaching was delivered via telephone, online, and face-to-face. Face-to-face coaching was available at limited locations and these participants also were able to interact with their coach by telephone and online. All participants enrolled in coaching were given the choice of using either or both telephone and online modalities. Online interactions included both emails from a participant to a coach and journal entries written by a participant to report on progress in coaching; all online correspondence occurred within a HIPPA-secure, password-protected website. Coaches were able to respond to both emails and journal entries.

Goals generally focused on one of the nine health-related areas previously outlined. They were typically set in 30-day increments using SMART format (eg, “I will lose 5 pounds in the next 30 days”). But the goal period/timeframe could be longer or shorter depending on the complexity of the goal and how frequently they interacted with their coach. Once a goal was identified, coaches and participants established action steps to support goal achievement (eg, limiting unhealthy foods to support weight loss, practicing breathing exercises to reduce stress). Supporting activities were most often set in 2 week increments, but could be of shorter duration when appropriate (eg, acquiring exercise equipment or healthy foods). Eligible participants were able to remain in the program as long as they continued to work on setting and achieving goals.

Coaching intervention characteristics were consistent with the components defined by the International Consortium for Health and Wellness Coaching [32], including creating an ongoing relationship with a coach, partnering of coach and individual in setting goals, incorporating self-discovery and active learning processes, and ongoing monitoring of and accountability for progress toward goal completion.

More specifically, the coaching philosophy was holistic and personalized to the individual, designed to facilitate behavior change through a one-to-one relationship with a coach. Coaches had a bachelor’s or master’s degree in psychology, nutrition, exercise physiology, nursing, or other health profession, and received extensive training in person-centered coaching strategies, cognitive behavioral techniques, positive psychology strategies, and other behavior change methods. Coaches used behavior change techniques to support participants in collaboratively setting goals and action plans, overcoming barriers, enhancing motivation, and assessing/building on progress. Quality of coaching interactions was monitored and evaluated; all coaches underwent 16 hours of training, passed a practicum, and participated in ongoing continuing training and routine quality assessments to assure that coaching protocols were adhered to.

### Measures

Data on gender and age, as well as information about health-related status and behaviors (weight, tobacco use, eating habits, stress) were collected during program registration. To better understand age-related trends, age ranges were collapsed into four groups: participants younger than 30 years, those aged between 30 and 50 years, those aged between 51 and 64 years, and those aged 65 years or older.

#### Coaching Modality

Coaching modality was identified by the types of interactions between coaches and participants documented within the coaching platform. Modality was classified into four groups: (1) online participants who were coached solely via the website, (2) mixed modality participants who worked with their coach by telephone and online, (3) telephone participants who interacted with their coach solely by phone, and (4) face-to-face participants, a combined group who held one or more face-to-face interactions with their coach and may or may not also have worked with their coach online and/or by telephone. Total interactions by method were computed. In all analyses, three online interactions were considered to equal one telephone or face-to-face coaching session; this ratio was derived from subject matter experts independent of the research team.

#### Goal Type

The coach used a standard list to document a participant’s goal. Categories included weight management, nutrition, fitness, tobacco cessation, stress management, diabetes management, cholesterol management, blood pressure management, back care, or “other.”

#### Goals Set and Completed

Within the coaching system, coaches documented each goal that was set and completed. The number documented within the system was used for analyses.

#### Action Steps Set and Completed

Also within the coaching platform, coaches documented each action step set and completed by participants, and the number documented within the system was used for analyses.

### Statistical Analysis

Descriptive statistics were generated and significance tests were conducted to test gender and age differences across various measures related to engagement and progress in coaching for participants who enrolled in coaching between January 2012 and August 2016. All analyses were conducted using SAS version 9.4.

#### Gender and Age Differences in Coaching Modality

Unadjusted multinomial logistic regression was used to model differences in coaching modality (electronic/Web/email, telephone, in-person, mixed) by gender. Linear regression with robust standard errors was used to assess differences in age by modality.

#### Gender and Age Differences in Goal Type

To assess who set what types of goals, each participant’s goal history was coded to determine if a certain goal type was set (eg, a value of “1” was assigned if a participant set that type of goal at any point in the program; otherwise “0” was assigned). Because participants could set more than one type of goal, separate unadjusted logistic regression models were used to assess the differences in this outcome by gender for each goal type. To assess differences in goal types set by age, mean age was compared for those participants who set a particular goal type (eg, weight management) versus those that never set that type of goal using a Welch test to account for unequal group variances.

#### Gender and Age Differences in Number of Goals, Action Steps, and Interactions

Intrapersonal variations in the number and type of coaching interactions as well as differences in total goals set and how goals were achieved through action steps were modeled as counts. To determine age and gender differences in the action steps set and completed, as well as goals set and completed, unadjusted negative binomial regression was used. Negative binomial regression models relax the assumption of equidispersion characteristic of a Poisson process.

#### Gender and Age Differences in Timing of Initial Goal Completion

We determined whether a member completed a goal within 30, 60, or 120 days rather than conduct survival analysis because our data source captured only time to completion for members completing goals. Separate uncontrolled logistic regressions were estimated with completion in 30, 60, or 120 days modeled as binary outcomes.

## Results

### Modality Preference

Gender differences in the type of coaching interactions chosen were found for all modalities (Table 1). Women were more likely than men were to choose online interactions and engage in face-to-face coaching sessions, whereas men were more likely to choose telephone sessions and engage with their coach via mixed modalities (a combination of telephone and online interactions). Similarly, age-related differences in the type of coaching interactions chosen were found for all comparisons except for online versus in-person interactions (Table 2).

**Table 1:** Gender differences in modality preference and type of goal set (N=35,271).

Modality and goal type		Male (n=8493)	Female (n=26,778)	Difference^a^	OR (95% CI)^b^	*P*
**Modality, predicted probability**						
	Online	0.63	0.71	–0.08		<.001
	Telephone	0.20	0.16	0.05		<.001
	In-Person	0.03	0.04	0.02		<.001
	Mixed	0.14	0.13	0.01		<.001
**Goal type, n (%)**^c^						
	Weight management	4464 (52.56)	16,596 (61.98)		0.68 (0.65-0.71)	<.001
	Fitness	1832 (21.46)	5747 (21.57)		1.01 (0.95-1.07)	.83
	Nutrition	1138 (13.40)	3985 (14.88)		0.86 (0.82-0.95)	<.001
	Stress management	576 (6.78)	2285 (8.53)		0.78 (0.71-0.86)	<.001
	Tobacco cessation	870 (10.24)	1657 (6.19)		1.73 (1.59-1.89)	<.001
	Cholesterol	352 (4.14)	738 (2.75)		1.53 (1.34-1.74)	<.001
	Blood pressure	407 (4.79)	639 (2.38)		2.06 (1.81-2.34)	<.001
	Diabetes	330 (3.88)	678 (2.53)		1.56 (1.36-1.78)	<.001
	Back care	206 (2.43)	328 (1.22)		2.01 (1.68-2.39)	<.001

^a^Differences were differences in predicted probabilities from multinomial logistic regression with bootstrapped standard errors.

^b^Odds ratios from unadjusted logistic regression.

^c^The percentage is derived from the total number of goals in each goal type set by each gender.

The average age of members preferring telephone coaching was oldest, approximately 16 years older than the average of those preferring online coaching and 10 years older than those choosing mixed telephone and online interactions.

### Type of Goal Set

Differences by gender were found for the types of goals participants chose to set except fitness (Table 1). Of particular note, women were more likely than men were to set goals to manage weight, whereas men were more likely than women were to set goals to quit tobacco or manage their diabetes. Although age differences were found for all goal types (Table 2), the greatest differences were that older individuals were more likely to set condition-related goals (eg, diabetes management, blood pressure management).

### Number of Goals, Action Steps, and Interactions

Results indicated no significant gender differences in goal setting; almost half of participants set one goal, with approximately 30% (10,528/35,333, 29.80%) setting two goals and slightly more than 20% (7904/35,333, 22.37%) setting three or more goals (Table 3). Genders differed in other aspects of the coaching process: women set and completed more action steps, and interacted more frequently with their coaches. Women set 12% more action steps than men did, had a mean of 21% more interactions than men did, and completed 12% more action steps than men did. Despite this increased activity among women, no differences by gender were found in overall number of goals goal completed by men and women.

Results indicated significant age-related differences as well (Tables 4 and 5), with older participants generally demonstrating more program activity across all age categories. This trend was apparent in the percentage of participants setting different numbers of goals; among those younger than 30 years, for example, more than 50% (2500/4653, 53.73%) set one goal and less than 15% (695/4653, 14.94%) set three or more goals, whereas among those aged 51 to 64 years, well over half set two or more goals (4842/8663, 55.89%) and 30% (1185/3911, 30.30%) of individuals aged 65 years and older set three or more goals. Significant differences were found among all age-related pairwise comparisons in number of goals set. Older participants set significantly more goals and action steps than their younger counterparts when comparing all age ranges. Coaching interactions peaked among those aged 51 to 64 years, with lower levels among both the older and younger groups. Similar to setting goals and action steps, completion of these coaching activities was highest among the oldest group of participants and decreased significantly at each age range.

### Differences in Timing of Initial Goal Completion

Regarding gender, the percentage of males and females completing their first goal within 30 days was approximately 10% (3544/35,271, 10.05%), whereas more than 80% (30,488/35,271, 86.43%) completed their initial goal within 60 days, and almost all participants completed them within 120 days (33,538/35,271, 95.09%) (Table 3). Statistically significant gender differences in the percentage of participants completing their initial goal emerged at 60 and 120 days, Age variations were also seen in the percentage of participants completing their initial goal within 30 days; all pairwise comparisons were significant, with older participants more likely to complete than younger participants were.

This trend continued for initial goal completion at 60 and 120 days, although it was not significant for all pairwise comparisons (Tables 4 and 5).

### Goal Completion by Goal Type

To further understand subgroup differences by goal type, the number of goals completed for each of the three most prevalent goal types (weight management, fitness, nutrition)—comprising approximately 80% of all goals set—was also compared. No gender differences were found in number of goals completed within specific goal types (Table 3). Age-related differences were found for all comparisons except those between individuals aged 51 to 64 years and 65 years or older working on fitness or nutrition goals, with individuals of older ages completing more goals within the specific goal types (Tables 4 and 5).

**Table 2:** Age differences in modality preference and type of goal set (N=35,333).

Modality and area of focus		Age (years)		Comparison^a^ age (years)		Difference (SE)		*P*	
		Mean (SD)		Mean^b^ (SD)					
**Modality**									
	Online		42.27 (11.78)						
	Telephone		58.26 (14.74)						
	In-person		41.72 (10.65)						
	Mixed		49.88 (14.42)						
	Modality comparison								
	Online vs telephone						15.99 (0.22)		<.001
	Online vs in-person						0.55 (0.65)		>.99
	Online vs mixed						–7.61 (0.24)		<.001
	Telephone vs in-person						16.54 (0.68)		<.001
	Telephone vs mixed						8.38 (0.31)		<.001
	In-Person vs mixed						–8.16 (0.68)		<.001
**Area of focus**^c^									
	Weight management		45.14 (13.62)		45.70 (14.50)				<.001
	Fitness		44.77 (13.87)		45.66 (14.01)				<.001
	Nutrition		44.56 (14.42)		45.62 (13.90)				<.001
	Stress management		46.30 (14.74)		45.40 (13.91)				.002
	Tobacco cessation		44.36 (12.93)		45.56 (14.06)				<.001
	Cholesterol		49.24 (13.15)		45.35 (13.99)				<.001
	Blood pressure		50.08 (14.39)		45.33 (13.95)				<.001
	Diabetes		58.27 (13.07)		45.09 (13.83)				<.001
	Back care		52.28 (16.04)		45.37 (13.93)				<.001

^a^Comparisons based on Welch test.

^b^Mean comparison age is the mean age of all participants not working on the designated goal type.

^c^Coaching area of focus includes all participants working on the designated goal type.

**Table 3:** Gender differences in program activity (N=35,333).

Program activity		Female	Male	OR/exp(β) (95% CI)^a^	*P*
**Number of goals set, n (%)**					
	1 goal	12,701 (47.43)	4175 (49.16)		
	2 goals	8062 (30.11)	2456 (28.92)		
	≥3 goals	6015 (22.46)	1862 (21.92)		
**Activity, mean (SD)**					
	Number of goals set	2.25 (2.48)	2.22 (2.57)	1.02 (0.98-1.04)	.13
	Number of action steps set	5.86 (12.45)	5.25 (11.32)	1.12 (1.07-1.17)	<.001
	Number of interactions	5.65 (11.60)	4.69 (10.25)	1.21 (1.16-1.25)	<.001
	Number of action steps completed	4.72 (11.94)	4.20 (10.82)	1.12 (1.06-1.19)	<.001
	Number of goals completed	1.23 (2.58)	1.19 (2.65)	1.04 (0.99-1.08)	.12
**Goals completed, mean (SD)**					
	Weight management	1.09 (2.25)	1.10 (2.26)	0.99 (0.97-1.02)	.77
	Nutrition	1.25 (2.87)	1.20 (2.62)	1.02 (0.97-1.08)	.43
	Fitness	1.21 (2.54)	1.16 (2.59)	1.02 (0.96-1.08)	.51
**First goal completed, n (%)**					
	Within 30 days	2644 (9.87)	900 (10.60)	0.92 (0.84-1.02)	.16
	Within 60 days	23,027 (85.96)	7461 (87.85)	0.85 (0.78-0.93)	<.001
	Within 120 days	25,401 (94.86)	8137 (95.81)	0.72 (0.70-0.93)	.001

^a^Odds ratio (OR) from unadjusted logistic regression for activity and days first goal completed within. Exponentiated coefficients (incident rate ratios) from unadjusted negative binomial regression for number of goals completed.

**Table 4:** Differences in program activity by age range (N=35,333).

Program activity		Age range (years)							
		<30		30-50		51-64		≥65	
**Number of goals set, n (%)**									
	1 goal		2500 (53.73)		8987 (49.64)		3821 (44.11)		1593 (40.73)
	2 goals		1458 (31.33)		5382 (29.72)		2555 (29.49)		1133 (29.97)
	≥3 goals		695 (14.94)		3737 (20.64)		2287 (26.40)		1185 (30.30)
**Activity, mean (SD)**									
	Number of goals set		1.85 (1.59)		2.12 (2.25)		2.48 (2.89)		2.81 (3.57)
	Number of action steps set		3.34 (7.08)		5.03 (11.05)		6.83 (13.82)		9.36 (16.96)
	Number of interactions		3.42 (7.99)		5.12 (10.79)		6.79 (13.41)		6.20 (11.37)
	Number of action steps completed		2.44 (6.65)		3.96 (10.54)		5.66 (13.29)		7.84 (16.24)
	Number of goals completed		0.76 (1.59)		1.09 (2.25)		1.49 (2.89)		1.83 (3.57)
**Goals completed, mean (SD)**									
	Weight management		0.66 (1.45)		0.97 (1.95)		1.30 (2.49)		1.74 (3.41)
	Nutrition		0.75 (1.73)		1.16 (2.38)		1.60 (3.76)		1.65 (3.81)
	Fitness		0.74 (1.62)		1.05 (2.38)		1.60 (3.16)		1.95 (2.94)
**First goal completed, n (%)**									
	Within 30 days		302 (6.49)		1678 (9.27)		958 (11.06)		621 (15.87)
	Within 60 days		4130 (88.76)		15,741 (86.94)		7331 (84.62)		3341 (85.43)
	Within 120 days		4462 (95.90)		17,279 (95.43)		8146 (94.03)		3713 (994.94)

**Table 5:** Comparison of differences in program activity by age range (N=35,333).

Program activity		≥65 vs							51-64 vs					30-50 vs	
		51-64	*P*	30-50	*P*	<30	*P*	30-50		*P*	<30	*P*	<30		*P*
**Activity, exp(β) (95% CI)**^a^															
	Number of goals set	1.14 (1.09-1.18)	<.001	1.33 (1.28-1.37)	<.001	1.52 (1.45-1.60)	<.001	1.17 (1.13-1.20)		<.001	1.34 (1.29-1.40)	<.001	1.15 (1.11-1.19)		<.001
	Number of action steps set	1.37 (1.26-1.49)	<.001	1.86 (1.72-2.01)	<.001	2.81 (2.54-3.09)	<.001	1.36 (1.28-1.44)		<.001	2.05 (1.88-2.22)	<.001	1.51 (1.40-1.62)		<.001
	Number of interactions	0.91 (0.85-0.98)	.01	1.21 (1.14-1.29)	<.001	1.82 (1.67-1.97)	<.001	1.33 (1.26-1.39)		<.001	1.98 (1.85-2.42)	<.001	1.49 (1.40-1.59)		<.001
	Number of action steps completed	1.39 (1.24-1.56)	<.001	1.98 (1.78-2.19)	<.001	3.21 (2.81-3.66)	<.001	1.43 (1.32-1.54)		<.001	2.31 (2.07-2.59)	<.001	1.62 (1.47-1.79)		<.001
	Number of goals completed	1.23 (1.13-1.35)	<.001	1.68 (1.55-1.83)	<.001	2.42 (2.18-2.68)	<.001	1.37 (1.29-1.45)		<.001	1.96 (1.80-2.15)	<.001	1.44 (1.32-1.56)		<.001
**Goals completed, exp(β) (95% CI)**^a^															
	Weight management	1.34 (1.22-1.47)	<.001	1.79 (1.64-1.95)	<.001	2.62 (2.35-2.93)	<.001	1.33 (1.42-1.23)		<.001	1.96 (1.78-2.15)	<.001	1.46 (1.34-1.60)		<.001
	Nutrition	1.03 (0.85-1.24)	>.99	1.42 (1.20-1.69)	<.001	2.18 (1.78-2.68)	<.001	1.38 (1.23-1.55)		<.001	2.12 (1.8-2.49)	<.001	1.53 (1.33-1.77)		<.001
	Fitness	1.22 (0.99-1.51)	.40	1.86 (1.54-2.25)	<.001	2.65 (2.11-3.33)	<.001	1.52 (1.31-1.77)		<.001	2.17 (1.78-2.64)	<.001	1.43 (1.20-1.69)		.001
**First goal completed, OR (95% CI)**^b^															
	Within 30 days	1.52 (1.31-1.76)	<.001	1.85 (1.62-2.11)	<.001	2.72 (2.24-3.30)	<.001	1.22 (1.09-1.36)		<.001	1.79 (1.49-2.15)	<.001	1.47 (1.24-1.75)		.001
	Within 60 days	1.07 (0.92-1.23)	>.99	0.88 (0.77-1.01)	.07	0.74 (0.63-0.88)	<.001	0.83 (0.75-0.91)		<.001	0.70 (0.60-0.81)	<.001	0.84 (0.74-0.97)		.005
	Within 120 days	1.19 (0.95-1.49)	.25	0.90 (0.72-1.11)	>.99	0.80 (0.61-1.06)	.21	0.75 (0.65-0.88)		<.001	0.67 (0.54-0.85)	<.001	0.89 (0.72-1.11)		>.99

^a^Exponentiated coefficients (incident rate ratios) from unadjusted negative binomial regression. Comparisons were produced using the SAS GENMOD procedure specifying the negative binomial distribution and LSMEANS statement with DIFF, ADJUST, and EXP options.

^b^Odds ratio (OR) from unadjusted logistic regression.

## Discussion

In this study of intergroup variations in coaching program participation, we found significant gender- and age-related differences in how people participate in and progress through a coaching program. Age-related variations encompassed all aspects of coaching activity, from initial choice of coaching modality (online, telephone) and goal type (eg, weight management, tobacco cessation) to goal completion as well as time to goal completion, whereas gender-related differences were demonstrated for all program activities except number of goals set and completed.

This research extends previous work indicating intrapersonal variation in program enrollment, retention, and completion. Prior research found gender differences in program engagement and retention in coaching programs [17-19]. Similarly, this study found that women were more likely to engage or interact with their coach than men were. In addition, we found that men and women differ in the modality by which they choose to interact with their coach, with women preferring online interactions, whereas men preferred other forms of interaction. Genders also differed in what they choose to address in coaching; women were more likely to set goals to manage weight, whereas men were more likely to set goals to quit tobacco or manage a condition such as diabetes. Women enrolled in coaching also were more actively involved, not just in interacting more frequently with their coach, but also in setting and completing more action steps. This finding suggests an opportunity to engage men differently in lifestyle change programs. Women and men, however, did not differ in number of goals set and completed.

This work extends past research examining age-related differences among participants in coaching programs, which found variations particularly in program retention and completion [17-19]. In addition to confirming age-related differences in program engagement, this study also found systematic age-related differences in all aspects of program activity. Participants preferring telephone coaching were, on average, 15 years older than those preferring to interact with their coach online. Age-related variations also were found among the types of goals participants chose to set, most notably with older participants being more likely to work on goals that support management or reduction of health-related risks such as elevated blood pressure, cholesterol, or diabetes. Additionally, all aspects of program progression—setting goals and action steps as well as completing them—saw increasing rates of activity with increasing age. In particular, goal completion increased with age across all goal types as well as within specific areas of focus (eg, weight management, nutrition, fitness). Similar age-related trends were seen in the percentage of participants completing their initial goal, with significant variation in completion at 30 days and some variation depending on age comparisons at 60 and 120 days postenrollment. Only among program interactions did the trend of increased activity with age vary somewhat, with number of coach-participant interactions peaking among those in the 51 to 64 years age group and declining somewhat among those 65 years and older.

These findings may offer new insights to help better design and target wellness promotion and interventions that lead to behavior change and health improvement. Results of this study underscore the importance of addressing intrapersonal differences. Starting with promotional materials, individuals of different ages and genders may respond more favorably to messaging tailored to their preferred areas of focus (eg, weight loss, tobacco cessation); alternatively, organizations could shift their messaging to entice enrollment in coaching for areas not currently utilized as heavily. Once enrolled in coaching, coaches may need to work more actively to engage men and younger participants in various aspects of the coaching process, providing additional support around setting and completing action steps to support goals with the knowledge that completing more action steps increases the likelihood of goal completion.

Finally, despite intrapersonal variation, coached participants continue to have much in common. For example, the majority of participants in coaching chose to work on weight management despite significant differences in other areas of focus. Likewise, increased rates of action step completion promote goal completion, regardless of gender or age. These findings strongly indicate that the process coaches use when working with participants should remain structured yet flexible, providing a framework setting the stage for behavior change while also personalizing the experience on the individual to meet his or her unique needs.

### Strengths and Limitations

This has many strengths, which include evaluating a large national sample with demographic and operational data from a diverse set of employers offering the same health coaching program to their employees. With these strengths, there are some limitations to point out.

First, results may only generalize to employer-sponsored health coaching programs and not to other types of wellness programs (non-employer sponsored program) or to other populations such as Medicare, etc. Additionally, this study included two key demographic metrics, age and gender, but did not include race or socioeconomic indicators because these were not collected. Information regarding chronic conditions was also not available for this study. Additional patterns and findings could be uncovered with additional demographic and condition-related data.

Health coaching programs offered may differ in the modalities delivered, length of treatment, etc. Thus, results may not generalize to other health coaching programs offered to employers. However, this program included the core elements defined by the International Consortium for Health and Wellness Coaching and should generalize to others meeting these standards.

### Future Directions

Expanding this work in several ways can widen its applicability within the coaching process. In this study, we explored how intrapersonal demographic factors influence variations in coaching participation and progress. Additional work is needed around psychological and behavioral factors and how they influence coaching participation and progress, as well as environmental and cultural factors within the worksite and beyond. Our findings, for example, suggest that if we can find new and different ways to engage younger participants, who may not yet feel the need for lifestyle change, we may inculcate healthy behaviors at a younger age and potentially reduce the need for people to address chronic health-related risks later in life. Alternatively, younger individuals may be more amenable to primarily digital programs and/or programming that incorporates social media. Supplemental work identifying these and other factors can provide a more holistic picture of the influencers of participation and progress in wellness programming.

Additionally, it will be important to connect this work to program outcomes beyond goal completion or program completion. Examination of health-related outcomes, such as weight loss and positive biometric changes, as well as the subjective appraisal of health are important to understanding the influence of intrapersonal variations on health status in addition to their influence of program participation and progression.

### Conclusions

Research in health coaching demonstrates it is a key intervention in health behavior change, and that the process of goal setting and achievement is foundational to the intervention’s success. The question of how to optimize coaching interventions, however, requires significant additional study. This study found significant intrapersonal variation in how people participate in and progress through a coaching program. Age-related variations were found in all aspects of coaching activity, from modality preference and initial choice of goal type (eg, weight management, tobacco cessation) to goal completion, whereas gender-related differences were demonstrated for all program activities except number of goals set and completed. These findings indicate that to maximize behavior change, coaches need to personalize the coaching experience to the individual.

## Abbreviations

IRB: Institutional Review Board
OR: odds ratio
